# Impact and burden of sickle cell disease in critically ill obstetric patients in a high dependency unit in Sierra Leone—a registry based evaluation

**DOI:** 10.1186/s12884-023-05888-9

**Published:** 2023-08-12

**Authors:** Milena Mortara, Momoh Sitta Turay, Sonia Boyle, Claudia Caracciolo, Sarjoh Bah, Henry Kargbo, Eva Hanciles, Valerie John-Cole, Ester Scapini, Roberto Benoni, Vishmi Dissanayake, Abi Beane, Rashan Haniffa, Adeniji O. Adetunji, Williamson Taylor, Luigi Pisani

**Affiliations:** 1 Department of Anesthesia and Intensive Care, University of Piemonte Orientale, Novara, Italy; 2 Princess Christian Maternity Hospital, University of Sierra Leone Teaching Hospitals Complex, Freetown, Sierra Leone; 3 Section of Operational Research, Doctors with Africa–Cuamm, Padova, Italy; 4 Department of Anesthesia and Intensive Care, Connaught Hospital, University of Sierra Leone, Freetown, Sierra Leone; 5 Anesthesia and Intensive Care Medicine, University of Bari, Bari, Italy; 6 Mahidol–Oxford Tropical Medicine Research Unit (MORU), Mahidol University, Bangkok, Thailand; 7 Intensive Care Unit, Miulli Regional Hospital, Acquaviva Delle Fonti, Italy

**Keywords:** Sickle cell disease, Low-resource settings, Obstetric, High dependency unit

## Abstract

**Introduction:**

Sickle cell disease (SCD) in pregnancy is associated with worse maternal and neonatal outcomes. There is limited available data describing the burden and outcomes of critically ill obstetric patients affected by SCD in low-income settings.

**Objectives:**

We aimed to define SCD burden and impact on mortality in critically-ill obstetric patients admitted to an urban referral hospital in Sierra Leone. We hypothesized that SCD burden is high and independently associated with increased mortality.

**Methods:**

We performed a registry-based cross-sectional study from March 2020 to December 2021 in the high-dependency unit (HDU) of Princess Christian Maternity Hospital PCMH, Freetown. Primary endpoints were the proportion of patients identified in the SCD group and HDU mortality. Secondary endpoints included frequency of maternal direct obstetric complications (MDOCs) and the maternal early obstetric warning score (MEOWS).

**Results:**

Out of a total of 497 patients, 25 (5.5%) qualified to be included in the SCD group. MEOWS on admission was not different between patients with and without SCD and SCD patients had also less frequently reported MDOCs. Yet, crude HDU mortality in the SCD group was 36%, compared to 9.5% in the non SCD group (*P* < 0.01), with an independent association between SCD group exposure and mortality when accounting for severity on admission (hazard ratio 3.40; 95%CI 1.57—7.39; *P* = 0.002). Patients with SCD had a tendency to longer HDU length of stay.

**Conclusions:**

One out of twenty patients accessing a HDU in Sierra Leone fulfilled criteria for SCD. Despite comparable severity on admission, mortality in SCD patients was four times higher than patients without SCD. Optimization of intermediate and intensive care for this group of patients should be prioritized in low-resource settings with high maternal mortality.

**Supplementary Information:**

The online version contains supplementary material available at 10.1186/s12884-023-05888-9.

## Introduction

Hemoglobin S (HbS) is a variant form of hemoglobin due to an abnormal beta globin chain. Homozygotes for this mutation (HbSS) as well as less common compound heterozygotes suffer from sickle cell disease (SCD), a severe form of anemia whose hallmarks are vaso-occlusive phenomena and hemolysis. Less common genetic variants associated with the disease are SCD is associated with premature death especially in low-income countries [1], while sickle cell trait is a benign carrier condition in which one allele of the beta globin gene carries the sickle hemoglobin mutation, producing hemoglobin AS (HbAS). Worldwide, the prevalence of SCD is highest in sub-Saharan Africa, as sickle cell trait condition has a protective effect against severe falciparum malaria [2]. In Sierra Leone the exact prevalence and incidence of SCD is unknown but the frequency of the trait is estimated at 20–25% [3].

People affected with SCD may require recurrent hospitalizations for acute pain crises, infections, cardiac problems, renal failure, and acute chest syndrome. Pregnant women with SCD experience these risks as well as vascular effects due to the gravid uterus and placenta. SCD in pregnancy is associated with infections, severe preeclampsia [4] and increased rates of cesarean delivery [5]. Pregnancies complicated by maternal SCD are also at increased risk of fetal complications such as increase in abortion, preterm labour, premature rupture of membranes, low birthweight, fetal distress in labour and increased perinatal mortality [6].

Despite the relevant prevalence and potential effects on mother and child, there is limited available data describing maternal burden and outcomes of critically ill obstetric patients affected by SCD in low income settings. A 2011 study reported how women with SCD suffer from a significant excess risk of dying in Jamaica, although it was not focused on critically ill patients and was limited to non-survivors case analysis [7]. There is scarce evidence on processes optimization to improve prevention or treatment of SCD-related complications in critically ill obstetric patients in low-resource settings. The aim of the current study is to define SCD burden in critically-ill obstetric patients and explore whether this condition significantly impacts outcomes in an urban referral hospital in Sierra Leone. The study leverages a recently developed cloud-based registry of critically ill patients in the largest maternal facility in the country [8]. We hypothesize that SCD burden is high in critically ill obstetric patients and independently associated with increased obstetrical complications and mortality.

## Methods

### Study design

We performed a registry-based prospective cross-sectional study from March 2020 to December 2021 in the high-dependency unit (HDU) of Princess Christian Maternity Hospital PCMH, Freetown, Sierra Leone. The HDU digital registry received ethical approval and a waiver of informed consent from the Sierra Leone Ethics and Scientific Review Committee in June 2020, with an extension granted in August 2022. (reference number 009/08/2022). The study was carried out in accordance with the declaration of Helsinki guidelines for medical research involving human subjects and the University of Sierra Leone ethical guidelines. The study is reported following the Strengthening of the reporting of Observational Studies in Epidemiology (STROBE) statement guidelines and checklists [9] (Table S1).

### Setting

PCMH is the largest maternity referral hospital in Sierra Leone, receiving about 9000 admissions and 6500 deliveries per year [8]. The HDU is a 8-bed critical care ward in which critically-ill women receive basic critical care interventions, such as close monitoring of vital signs and renal output, intravenous fluid and vasopressor therapy management, blood transfusions, oxygen, antibiotics, essential point of care laboratory tests and pain management [10, 11]. Invasive mechanical ventilation, renal replacement therapy and other advanced organ support techniques were not available in this setting during the study period. The maternal mortality rate in Sierra Leone is the highest in the world with 1,120 mothers dying in every 100,000 live births, although a recent nation-wide survey reported this figure to be 510 per 100,000 live births [12]. Hospital maternal mortality rate during the study was 1049 deaths per 100.000 live births.

### Population

Patients were included in the study if admitted to the HDU for any cause during the observation period. The sickle cell disease group was assigned to a patient whenever this condition was flagged in the patient hospital record by the treating team and thus captured by the electronic HDU registry. The registry code for SCD thus included any of the following: (i) patients patients being admitted with a clinical complication of SCD (such as “sickle cell crisis” or “acute chest syndrome”) as reason for admission; (ii) patients with self-reported SCD as self- reported comorbidity on the parturient medical history, even if this was not the main reason for admission; (iii) patients having a positive point of care laboratory test for SCD (rapid detection test without differentiation of genotypes). The current registry coding for SCD did not allow to differentiate between these three conditions. Patients with incomplete records, i.e. with missing admission diagnosis or outcome data were planned to be excluded from analysis. The non-SCD group was defined as all other patients admitted to the HDU during study period.

### Registry structure and data collection procedures

The HDU in PCMH is part of the Critical Care Asia Africa (CCAA) federated network of registries, an international initiative enabling continuous evaluation of critical care services in low and middle income countries. Registry infrastructure and quality control processes were detailed in previous publications [13–15]. In brief, a dedicated data collector entered daily data on consecutive admissions on a secure cloud-based mobile or desktop portal. The clinical information was directly extracted from existing paper-based records and monitoring charts within the HDU. Validation of reporting and the opportunity for technical support were assured by regular contact with the registry platform curating team.

### Data collection

Data collected on admission included demographic data, reported reason for hospital and for HDU admission, comorbidities, surgical status, the presence of a maternal direct obstetric complication (MDOCs), ongoing treatments for malaria or other infections. Vital signs were collected at admission, at 24 h and at discharge; they included pulse oximetry oxygen saturation (SpO_2_), heart rate, respiratory rate, temperature, neurological status, systolic and diastolic arterial blood pressure, urinary output. Point of care laboratory measurement included glucose level and hemoglobin level. Total number of blood units transfused was recorded at discharge but only available in patients admitted after October 2021. Severity on admission was stratified through the Maternal Early Obstetric Warning Score (MEOWS; Table S2) [16]. Patient outcomes were recorded at HDU discharge.

### Study definitions

The MEOWS uses core physiological parameters such as systolic and diastolic blood pressure, heart rate, respiratory rate and temperature to identify critical obstetric patients. The MEOWS ranges from 0 to 10 points, with a green color code granted with a total of 1–2, a yellow color code for total 3–5, and a red color code attributed to every patient with > 5 points or any danger sign. (Table S2). The AVPU coma scale was used to rapidly assess the patient’s mental status in four categorical classes: alert (A), response to verbal stimuli (V), response to painful stimuli (P), unresponsiveness (U). Respiratory distress was defined as a respiratory rate on admission > 30 breaths per minute and/or a SpO_2_ in air < 92% and/or when acute respiratory failure was recorded as the reason for admission.

Eight different maternal direct obstetric complications (MDOCs) were recorded, namely ante-partum hemorrage (APH), post-partum hemorrage (PPH), uterine rupture, puerperal sepsis, obstructed labour, severe pre-eclampisa or eclampsia, complications of abortion and ectopic pregnancy [17, 18].

### Study endpoints

The co-primary endpoints were the proportion of patients identified in the SCD group and the crude HDU mortality of the SCD group compared to the non-SCD group. Secondary endpoints included meaningful clinical variables such as: severity of illness at admission, frequency of MDOCs and respiratory distress, hemoglobin levels, number of blood transfusions, malaria status on admission, HDU length of stay, organ support in terms of oxygen therapy and vasopressors.

### Statistical analysis

Due to the descriptive nature of this analysis we did not perform a formal sample size calculation – instead, all available patients from registry inception were included in the study. Demographic data and outcomes were summarized as medians (interquartile range) for continuous variables and as frequencies (percentage) for categorical variables. In the case of normally distributed, continuous variables were compared between patients with and without SCD using t–test or ANOVA. When not considered normally distributed, continuous variables were compared between groups with Mann–Whitney U test or Kruskal–Wallis test, as appropriate. Categorical variables were compared between groups by chi–square analysis. Missing data imputation was not performed.

The association between the SCD group with HDU mortality as a time–to–event was analyzed with a Cox regression model, reporting the hazard ratio with 95%–confidence interval (CI). The SCD group was used as the grouping variable, while MEOWS (continous variable) was entered as covariate expressing the severity on admission based on physiological parameters. All analyses were performed using a two–sided superiority hypothesis test, with a significance level of 0.05 and presented with two–sided 95%–CI. No corrections were performed for multiple comparisons across secondary clinical outcomes. Analyses were performed using software R (version 4.0.2, R Core Team, 2016, Vienna, Austria).

## Results

### Case-mix

Of a total of 497 patients included in the HDU registry in the study period, 25 (5.5%) qualified to be included in the SCD group. No patients were excluded due to missing data. Baseline characteristics of patients with SCD as compared to the patients admitted to HDU without SCD are detailed in Table 1. Patients with SCD had comparable demographic characteristics to those without SCD, with an average age at admission of 25 years old. Patients with SCD were less frequently post-operative patients (16% vs. 49%; *P* = 0.002) with cesarean section being the most frequent surgery performed followed by laparotomy (Table 1). Time elapsed from hospital entry to HDU admission was not significantly different between the two groups, with roughly 70% of patients being referred to HDU within 24 h of hospital admission.

**Table 1:** Patients baseline case-mix characteristics

	**All patients (*n* = 497)**	**SCD group (*n* = 25)**	**No-SCD group (*n* = 472)**	***P*-value**
**Age (years)**	26 (21, 30)	23 (21, 28)	26 (21, 30)	0.417
**Occupation status**				
Employed	167 (33.6)	8 (32.0)	159 (33.7)	1
Unemployed	113 (22.7)	8 (32.0)	105 (22.2)	0.373
Homemaker	207 (41.6)	9 (36.0)	198 (41.9)	0.704
**Type of admission**				
Non operative	260 (52.3)	21 (84.0)	239 (50.6)	**0.002**
Operative	237 (47.7)	4 (16.0)	233 (49.4)	**0.002**
Emergency surgery	96 (40.5)	1 (25.0)	95 (40.8)	0.648
**Reason for admission (Medical)**				
Anemia	29/260 (11.2)	3/21 (14.3)	26/239 (10.9)	0.714
Acute respiratory failure	28/260 (10.8)	2/21 (9.5)	26/239 (10.9)	1
Malaria	26/260 (10.0)	4/21 (19.0)	22/239 (9.2)	0.143
Convulsive syncope	12/260 (4.6)	1/21 (4.7)	11/239 (4.6)	1
**Reason for admission (Surgical)**				
Cesarean section	76/237 (32.1)	3/4 (75.0)	75/233 (32.2)	1
Laparotomy	6/237 (2.5)	0	6/233 (2.6)	1
Hysterectomy	4/237 (1.7)	0	4/233 (1.7)	1
Evacuation of uterus	3/237 (1.3)	1/4 (25.0)	3/233 (1.3)	1
**MDOCs on admission**				
None reported	148/495 (29.9)	17/23 (73.9)	131/472 (27.8)	** < 0.01**
APH	40/495 (8.08)	0	40/472 (8.47)	0.243
PPH	125/495 (25.3)	3/23 (13.0)	122/472 (25.8)	0.257
Prolonged/obstructed labour	8/495 (1.62)	0	8/472 (1.69)	1
Complication of abortion	3/495 (0.606)	0	3/472 (0.636)	1
Pre eclampsia/Eclampsia	112/495 (22.6)	3/23 (13.0)	109/472 (23.1)	0.385
Puerperal sepsis	24/495 (4.85)	0	24/472 (5.08)	0.618
Ectopic pregnancy	11/495 (2.22)	0	11/472 (2.33)	1
Ruptured uterus	24/495 (4.85)	0	24/472 (5.08)	0.618
**Comorbidities**				
None (No comorbidity)	444 (89.3)	9 (36.0)	435 (92.2)	** < 0.01**
AIDS or HIV	10 (2.0)	2 (8.0)	8 (1.7)	0.085
Asthma	1 (0.2)	0	1 (0.2)	1
Diabetes	4 (0.8)	0	4 (0.8)	1
Hepatic disease	1 (0.2)	0	1 (0.2)	1
Hypertension	20 (4.0)	0	20 (4.2)	0.614
Renal failure	4 (0.8)	0	4 (0.8)	1
Respiratory disease, severe/moderate	1 (0.2)	0	1 (0.2)	1
Tuberculosis	1 (0.2)	0	1 (0.2)	1
**Time from hospital to HDU admission**				
Less than 24 h	379 (76.3)	18 (72.0)	361 (76.5)	0.786
More than 24 h	118 (23.7)	7 (28.0)	111 (23.5)	0.786

*Abbreviations:* *SCD* Sickle cell disease, *APH* Antepartum hemorrhage, *PPH* Vipost-partum hemorrhage, *AIDS* Acquired immunodeficiency syndrome

**Fig. 1:**
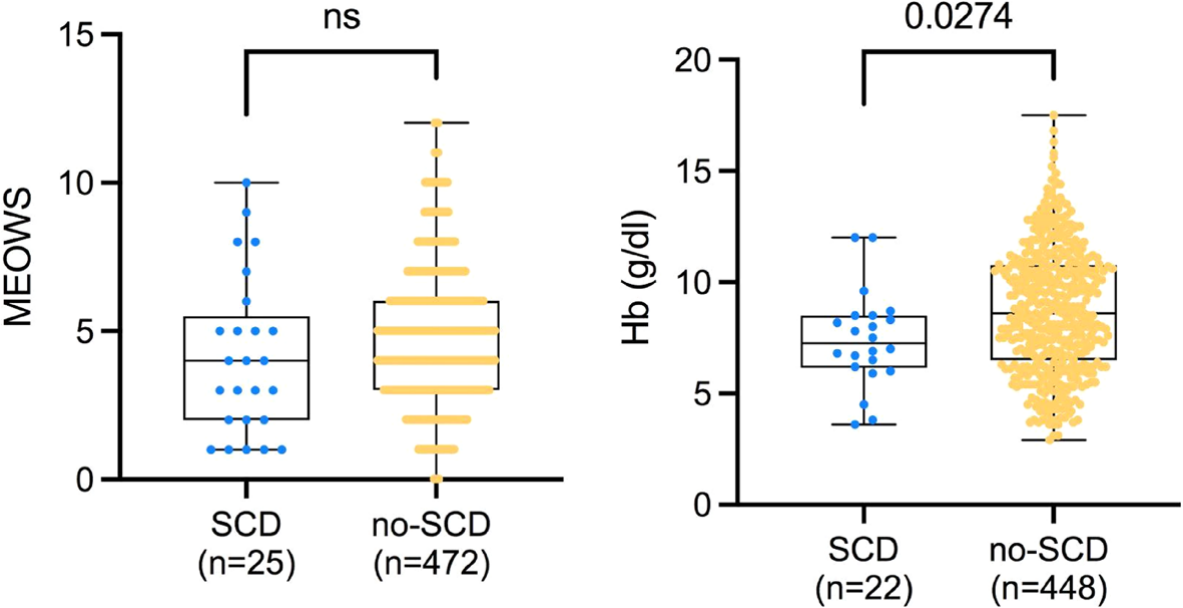
Boxplot of modified early obstetric warning score and hemoglobin levels in patients with SCD as compared to patients without SCD. *P*-Value was computed through Mann–Whitney U test. Abbreviations: ns, non significant; MEOWS, modified early obstetric warning score; Hb, hemoglobin

**Fig. 2:**
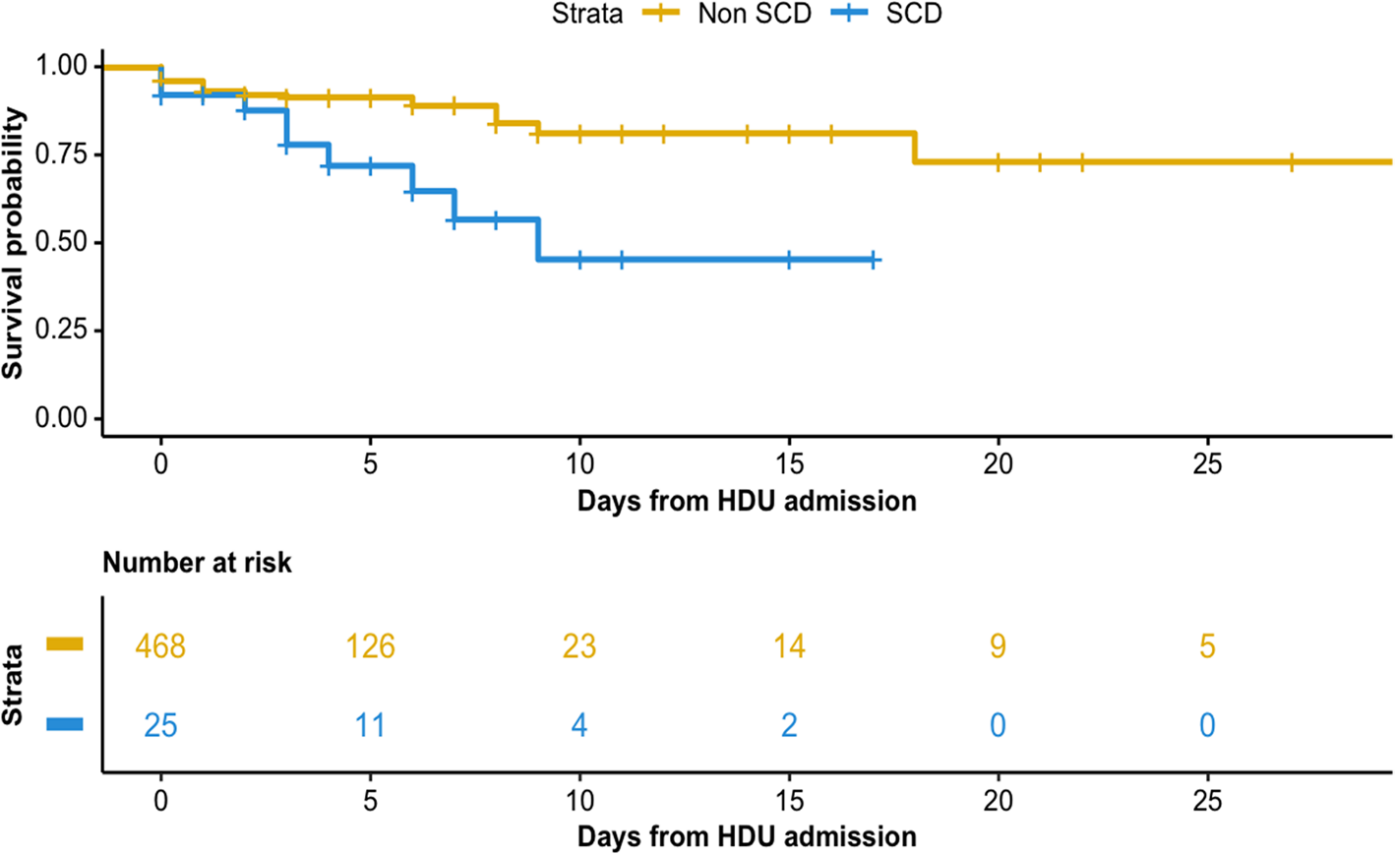
Survival curve for the SCD group as compared to the general HDU population. Abbreviations. SCD, sickle cell disease group; HDU high dependency unit

In the SCD subgroup, a major direct obstetric complication was reported in fewer cases than in the non-SCD group (26.1% versus 72.2%, *p* < 0.01), with PPH and pre-eclampsia/eclampsia being the MDOCs reported. SCD patients were more anemic, with capillary Hb levels of 7.2 gr/dl in the SCD group compared to 8.6 g/dl in the non-SCD group (*p* = 0.02) (Fig. 1). The severity level on admission was comparable with at least one red alert on the MEOWS recorded in > 75% in both groups (Table 2) and a total MEOWS of 4 (2–5) and 5 (3–6) in the SCD and non-SCD groups, respectively (*P* = 0.09; Fig. 2). The main reasons for admission in the SCD group were anemia, severe malaria, respiratory distress, and eclampsia. Acute respiratory failure as a reason for admission was observed in one out of ten patients in the SCD group, with no difference compared to the general population of critically-ill obstetric patients.

**Table 2:** Clinical parameters on admission

	**All patients (*n* = 497)**	**SCD group (*n* = 25)**	**No-SCD group (*n* = 472)**	***P*-value**
**Severity of illness**				
MEOWS	5 (3, 6)	4 (2, 5)	5 (3, 6)	0.089
Number of red alerts	407 (81.9)	19 (76.0)	388 (82.2)	0.432
Number of yellow alerts	404 (81.3)	18 (72.0)	386 (81.8)	0.221
Respiratory distress, yes	74 (14.9)	3 (12.0)	71 (15.0)	1
**Vitals on admission**				
SpO_2_, %	97 (95, 98)	97 (94, 98)	97 (95, 99)	0.327
FiO_2_, %	40 (22, 40)	40 (36, 40)	40 (22, 40)	0.963
SpO_2_/FiO_2_	242 (223, 289)	248 (242, 269)	242(220, 296)	0.406
Systolic BP, mm Hg	123 (105, 142)	122 (106, 132)	123 (105, 143)	0.547
Diastolic BP, mm Hg	77 (62, 94)	68 (61, 81)	78 (62, 94)	0.086
Respiratory rate, breaths per minute	30 (26, 38)	32 (28, 45)	30 (26, 37)	**0.042**
Heart rate, beats per minute	110 (98, 126)	100 (92, 112)	110 (98, 127)	**0.048**
Temperature, °C	36.8 (36.6, 37.3)	36.7 (36.5, 37.4)	36.8 (36.6, 37.3)	0.983
AVPU condition				
Alert	228 (45.9)	18 (72.0)	210 (44.5)	**0.012**
Voice	112 (22.5)	6 (24.0)	106 (22.5)	1
Pain	98 (19.7)	0	98 (20.8)	**0.007**
Unresponsive	58 (11.7)	1 (4.0)	57 (12.1)	0.340
**Laboratory**				
Capillary blood glucose, mmol/l	7.7 (5.8, 10.3)	5.7 (5.2, 6.6)	7.8 (5.9, 10.5)	**0.004**
Hemoglobin levels, g/dl	8.5 (6.5, 10.7)	7.3 (6.3, 8.5)	8.6 (6.5, 10.7)	**0.028**

*Abbreviations:* *SCD* Sickle cell disease, *MEOWS* Modified Early Obstetric Warning Score, *SpO2* Hemoglobin saturation from pulse oxymetry, *FiO*_*2*_ Fraction of inspired oxygen, *BP* Blood pressure, *AVPU* Alert, voice, pain, unresponsiveness score

### Association with HDU mortality

**Fig. 3:**
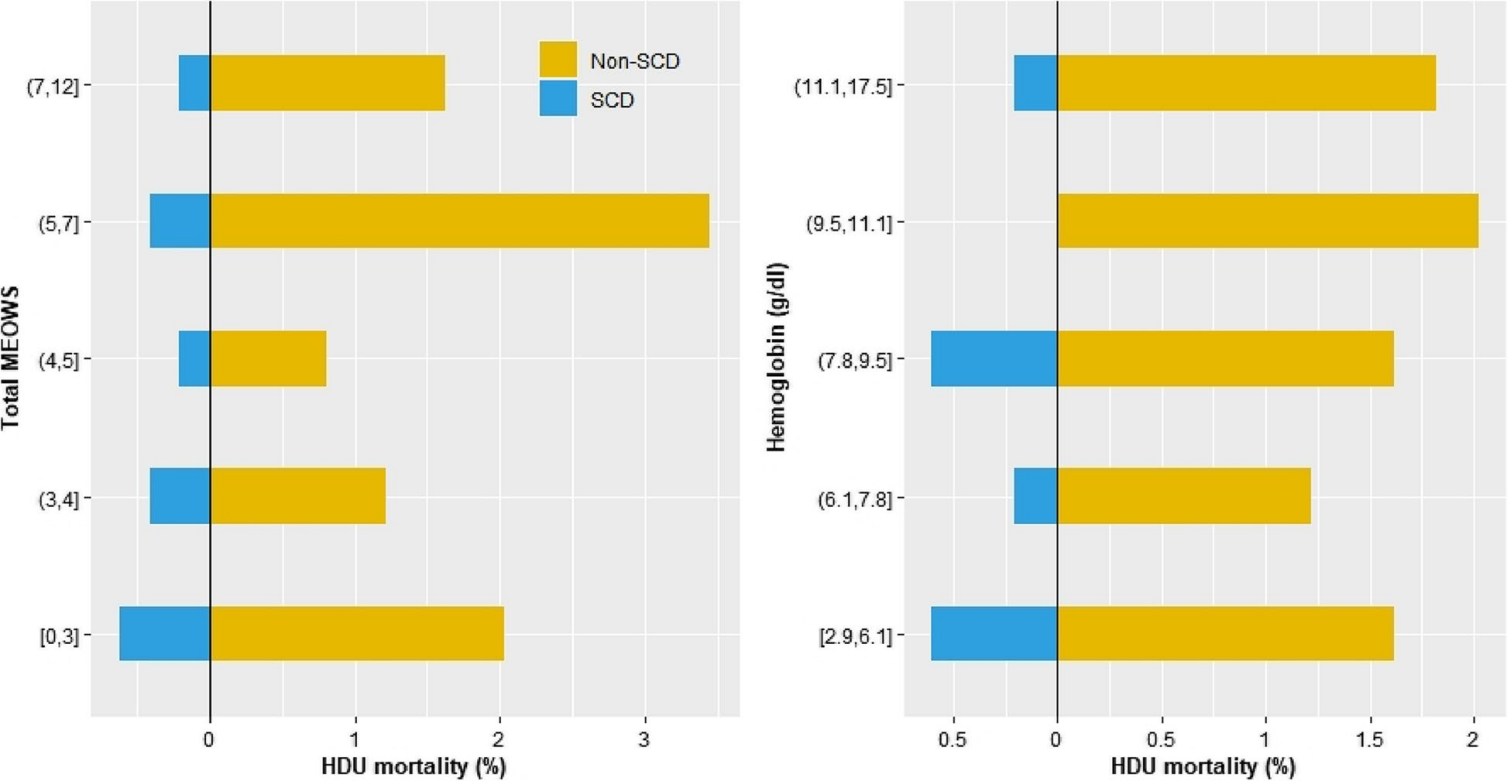
Divergent bar plot showing the crude HDU mortality rate stratified by maternal early obstetric warning score and hemoglobin levels. Abbreviations: SCD, sickle cell disease; HDU, high dependency unit; MEOWS, modified early obstetric warning score

Crude HDU mortality was higher in the SCD group compared to the group without (36% vs. 9%, *p* < 0.001; Table 3). Overall, SCD affected one in five HDU deaths. Having SCD on admission was associated with an increased risk of death in HDU after adjustment for the severity level on admission expressed by MEOWS (HR for SCD group 3.40; 95%CI 1.57—7.39; *P* = 0.002, Fig. 2 and model details in Table S3). Death in HDU was observed for SCD patients also at low levels of severity on admission and with normal hemoglobin levels (Fig. 3)*.* Patients with SCD had a tendency to longer median length of stay in both survivors and non survivors, although these findings did not reach statistical significance.

**Table 3:** Patient outcomes

	**All patients (*n* = 497)**	**SCD group (*n* = 25)**	**No-SCD group (*n* = 472)**	***P*-value**
**Death in HDU**	54/494 (10.9)	9/25 (36.0)	45/469 (9.5)	** < 0.001**
**Time from HDU admission to death, hours**	13 (2, 69)	74 (34, 140)	9 (2, 51)	0.090
**Length of stay in HDU for survivors, days**	3.1 (1.8, 4.8)	4.5 (2.7, 8.2)	3.0 (1.8, 4.7)	0.068
**Discharge destination for survivors**				
Home	65/440 (14.8)	2/16 (12.5)	63/424 (14.9)	1
Ward	355/440 (80.7)	12/16 (75.0)	343/424 (80.9)	0.524
Transfer for specialist care	6/440 (1.3)	1/16 (6.3)	5/424 (1.2)	0.200
Referral to ICU or other hospital	13/440 (2.9)	1/16 (6.3)	12/424 (2.8)	0.386
Other	1/440 (0.2)	0	1/424 (0.2)	1

*Abbreviations:* *SCD* Sickle cell disease, *HDU* High dependency unit, *ICU* Intensive care unit

### Management features

Organ support features in the first 24 h for patients with and without SCD are detailed in Table 4. Oxygen therapy was required by 36% of SCD patients and by 14% of the non-SCD group (*P* = 0.002). Antibiotic therapy was prescribed in 9 out of 10 patients in both groups, while SCD were three times more prone to receive anti-malarial treatment than patients without SCD. Vasoactive treatment was rarely required in the SCD group, yet reported in one fourth of patients without SCD, mainly for the management of hypertension in eclamptic patients. The data on total blood transfusions received during HDU stay was incomplete and only available for 14/25 SCD patients. Eleven out of 14 patients (79%) received a blood transfusion, with a median of 1 (1–4) units of whole blood per patient administered. No transfusion data was available for the non-SCD group.

**Table 4:** Management features

	**All patients (*n* = 497)**	**SCD group (*n* = 25)**	**No-SCD group (*n* = 472)**	***P*-value**
**Oxygen therapy**	73 (14.7)	9 (36.0)	64 (13.6)	**0.002**
**Vasoactive treatment**				
Vasopressors	40 (8.0)	2 (8.0)	38 (8.1)	1
Antihypertensives	121 (24.3)	1 (4)	120 (25.4)	1
**Antihypertensive agent**				
Intravenous hydralazine	8 (1.8)	0 (0.0)	8 (1.9)	1
Oral nifedipine	79 (18.3)	0 (0.0)	79 (18.2)	0.268
Any other	88 (20.4)	2 (8.0)	86 (20.1)	1
**Urinary catheter**	203/432 (46.9)	8/22 (36.4)	195/410 (47.4)	0.305
**Antimicrobial use**	445 (89.5)	22 (88.0)	423 (89.6)	0.737
**Of which, antimalarial therapy**	41/445 (9.5)	7/22 (31.8)	34/423 (7.2)	** < 0.01**

*Abbreviations:* *SCD* Sickle cell disease

## Discussion

The main findings from this study were: (1) one in 20 critically ill obstetric patients referred to an obstetric HDU in a limited resource setting had assigned a SCD code; (2) these patients were mainly non-operative and had comparable severity on admission to patients without SCD; (3) yet SCD patients suffered from a four-fold higher HDU mortality, with SCD affecting one in five HDU deaths; (4) the association between group exposure and mortality was independent of baseline severity (5) context-specific HDU management for SCD patients comprises oxygen therapy, transfusions and antimicrobial therapy.

In our study one out of twenty patients admitted to the HDU had a history of SCD or was suffering from a complication of this disease. Studies reporting on the prevalence of SCD in obstetrical critical patients are lacking, as is robust local epidemiological data in Sierra Leone regarding prevalence of SCD in adults. In the United States, SCD complicates one out of 100 pregnancies, but does account for 1% of all maternal deaths [19]. Data on the frequency of SCD among HDU patients must be cautiously interpreted taking into account two context-specific considerations. Firstly, it is not very common for a SCD patient to become a mother, since 50 to 90% of children affected by SCD in Africa die before the age of 5 [20]. Secondly, being the study hospital a large referral hospital for complex obstetric cases, the number of patients with SCD may have been higher than in other settings. Yet, our study raises an alarming flag on the potential weight of SCD complications in pregnancy in the critically ill obstetric populations in limited resource settings with high maternal mortality.

SCD patients had similar baseline characteristics and management features to the general HDU population except for the need of oxygen, which was predictably higher in the SCD group. This can be attributed most likely to episodes of vaso-occlusive phenomena, pulmonary infections and severe anemia. Hemoglobin levels were, as expected, significantly lower in the SCD population. On the other hand, hemorrhagic complications such as APH and PPH were less common in SCD women, in line with previous literature [19, 21, 22]. This finding is probably attributable to the hypercoagulability state associated with SCD. In fact, vaso-occlusive crises as well as infections and thromboembolic events are well-known peripartum complications described in SCD making the low frequency of cesarean sections an unexpected finding. Malaria was a common condition in our cohort, a plausible finding as only SCT but not SCD is known to have a protective effect against the disease. On the contrary, the malaria infection can be accompanied by severe complications when affecting individuals with SCD [23]. Defining this burden was a relevant finding in our study, as early diagnosis and supportive treatment of malaria complications in patients with SCD may impact patient outcomes.

Our findings extend previous studies reporting that SCD in pregnancy increases the risk of maternal death [24–27]. Overall, crude mortality rate was extremely high in this setting with one in three patients with SCD dying in the HDU, as compared to one in ten patients in the non SCD group. It is important to note how SCD patients represented only 5% of admissions but almost 17% of total deaths. A 2011 study from Jamaica examined a sample of 42 deaths occurring in SCD patients over a 10-years-long period, in a setting with greater availability of diagnostic and treatment options[7]. In that study, SCD deaths suffered blood disorder, cardiovascular diseases and higher rates of post-partum complications – and more often died in an ICU. The study reported a significant excess risk of dying, underscoring the urge of further exploration of possible pathophysiological mechanisms to inform appropriate interventions. Our findings extend those from Asnani et al., describing how also in critically ill patients there is an increased probability of death which is independent from the baseline severity [7]. In our study, patients with SCD died roughly 48 h later than women in the non-SCD group, a time gap that may be attributed to the pathophysiology of the deadly cardiovascular events often seen in SCD, although we have no patient-level data to discern the exact causes of death.

A recent large cross-sectional study from the United States assessing outcomes of acute SCD admissions with black race suggested prenatal anemia to be a possible mediator associated with pregnancy risk in individuals with SCD [28]. In our cohort anemia as an admission criteria was equally prevalent among the two groups, and we could not distinguish between prenatal anemia versus acute hemorrhagic conditions. Yet, the hemoglobin level was markedly lower in the SCD group, in line with the hypothesis endorsing prenatal anemia as a mediator for adverse pregnancy outcomes.

The evidence for poor outcomes in this group of patients calls for specific optimization and escalation of care focusing on the life-threatening SCD complications. Patients with suspected acute chest syndrome require transfusions, antibiotics, adequate analgesia and and may need enoxaparin at therapeutic dosage in case of thromboembolism [29]. In low-resource settings the consistency of drug's and blood availability is often a problem, and sporadic scarcity of life-saving treatments may contribute to worsen mortality.

A major management issue is the prompt access to blood transfusions. Raising hemoglobin levels is recommended by current guidelines [30, 31], with the recommendation to avoid increasing Hb beyond 10 g/dl in patients receiving a transfusion, due to concerns with whole-blood viscosity-related complications [32]. Immediate and continuous availability of blood products from the blood bank is a limiting factor in many African hospitals [33]. According to these findings, the implementation of strategies which lead to reliable blood supply in SCD patients to maintain Hb levels between 7 and 9 g/dl needs to become a primary concern. Moreover, as women with SCD have a steady state Hb level that is lower than average, routinary Hb screening in antenatal clinics may contribute to flag potential cases. This is even more important as prenatal anemia may be a mechanistic driver for organ dysfunction and complications associated with abnormal placentation [28]. In addition, the availability of reliable diagnostic methods based on electrophoresis or validated rapid detection kits should increase to allow correct diagnosis and phenotyping of SCD.

Our study has several strengths. It represents the first cloud-based registry-derived output from an obstetric HDU in a low-income setting. The registry structure allowed for a precise reporting of consecutive patient episodes, with a data collector dedicated to daily data entry and a registry infrastructure with inbuilt quality assurance features. Outcome follow-up at HDU discharge was completed for all patients, with an exact understanding of mortality and time-dependent endpoints.

The study also presents several limitations; the data collection was limited to obstetric HDU patients, therefore no information on neonatal outcomes was available. SCD diagnosis was acquired from self-reported anamnesis, patient records or clinically at HDU entry, and no electrophoresis confirmatory methods were available. Importantly, due to the unique coding for SCD we also lacked data on numbers for each defining category. Thus we cannot exclude potential miscategorization or the missing of patients affected by SCD with no prior knowledge. This limitation will be mitigated in the future by the implementation of a rapid detection test for SCD on all primigravidas. The outcomes were limited to patient HDU stay in immediate postpartum and we lacked the data to calculate a HDU-specific maternal mortality ratio. We also consider our study affected by a selection bias, since it was led in a large referral hospital, thus the findings cannot be generalized to district hospitals or different healthcare settings. Finally, this was a registry-based pragmatic study, with the systematic unavailability of some important variables conventionally used to specifically describe obstetric and SCD cohorts (e.g. parity and gravidity). This limited the patient-level characterization of conditions such as acute chest syndrome and other SCD related complications.

## Conclusions

In this registry-based prospective study we show how SCD burden in critically ill obstetric patients is significant and is associated with poor outcomes, despite a similar severity on admission to patients without SCD. These findings call for the optimization of intermediate and intensive care for SCD patients, to be prioritized in low-resource settings with high maternal mortality.

### Supplementary Information

**Additional file 1. **

## Data Availability

The datasets generated during and analyzed during the current study are not publicly available due to registry data sharing policies but are available from the corresponding author on reasonable request to the Critical Care Asia Africa (CCAA) data access committee. For further information and access to the data, please contact the CCAA data access committee (DAC@nicslk.com) and quote the manuscript, your institution and provide return correspondence information.
