# Supplementary material for: Impact and burden of sickle cell disease in critically ill obstetric patients in a high dependency unit in Sierra Leone—a registry based evaluation
Source: BMC Pregnancy Childbirth. 2023 Aug 12;23:580. doi: 10.1186/s12884-023-05888-9 (PMC10422830; doi:10.1186/s12884-023-05888-9)
Supplement: Supplementary file 1 — Additional file 1. [file 12884_2023_5888_MOESM1_ESM.docx]

**SUPPLEMENTARY MATERIAL to**

**Impact and burden of Sickle Cell Disease in critically ill obstetric patients in a high dependency unit in Sierra Leone - a registry based evaluation**

**Table S1. STROBE checklist**

|  | Item No | Recommendation | **Page**  **No** |
| --- | --- | --- | --- |
| Title and abstract | 1 | (*a*) Indicate the study’s design with a commonly used term in the title or the abstract | **1** |
|  |  | (*b*) Provide in the abstract an informative and balanced summary of what was done and what was found | **2** |
| Introduction | | | |
| Background | 2 | Explain the scientific background and rationale for the investigation being reported | **3** |
| Objectives | 3 | State specific objectives, including any prespecified hypotheses | **3** |
| Methods | | | |
| Study design | 4 | Present key elements of study design early in the paper | **4** |
| Setting | 5 | Describe the setting, locations, and relevant dates, including periods of recruitment, exposure, follow-up, and data collection | **5** |
| Participants | 6 | (*a*) Give the eligibility criteria, and the sources and methods of selection of participants | **5-6** |
| Variables | 7 | Clearly define all outcomes, exposures, predictors, potential confounders, and effect modifiers. Give diagnostic criteria, if applicable | **7** |
| Data sources/ measurement | 8* | For each variable of interest, give sources of data and details of methods of assessment (measurement). Describe comparability of assessment methods if there is more than one group | **7** |
| Bias | 9 | Describe any efforts to address potential sources of bias | **Not done** |
| Study size | 10 | Explain how the study size was arrived at | **Not done** |
| Quantitative variables | 11 | Explain how quantitative variables were handled in the analyses. If applicable, describe which groupings were chosen and why | **7** |
| Statistical methods | 12 | (*a*) Describe all statistical methods, including those used to control for confounding | **7** |
|  |  | (*b*) Describe any methods used to examine subgroups and interactions | **Not applicable** |
|  |  | (*c*) Explain how missing data were addressed | **Not applicable** |
|  |  | (*d*) If applicable, describe analytical methods taking account of sampling strategy | **Not applicable** |
|  |  | (*e*) Describe any sensitivity analyses | **Not applicable** |
| Results | | | |
| Participants | 13* | (a) Report numbers of individuals at each stage of study—eg numbers potentially eligible, examined for eligibility, confirmed eligible, included in the study, completing follow-up, and analysed | **9** |
|  |  | (b) Give reasons for non-participation at each stage | **Not applicable** |
|  |  | (c) Consider use of a flow diagram | **Not included** |
| Descriptive data | 14* | (a) Give characteristics of study participants (eg demographic, clinical, social) and information on exposures and potential confounders | **6** |
|  |  | (b) Indicate number of participants with missing data for each variable of interest | **NA** |
| Outcome data | 15* | Report numbers of outcome events or summary measures | **9** |
| Main results | 16 | (*a*) Give unadjusted estimates and, if applicable, confounder-adjusted estimates and their precision (eg, 95% confidence interval). Make clear which confounders were adjusted for and why they were included | **9, 10, Supplement** |
|  |  | (*b*) Report category boundaries when continuous variables were categorized | **9,10** |
|  |  | (*c*) If relevant, consider translating estimates of relative risk into absolute risk for a meaningful time period | **Not applicable** |
| Other analyses | 17 | Report other analyses done—eg analyses of subgroups and interactions, and sensitivity analyses | **9,10** |
| Discussion | | | |
| Key results | 18 | Summarise key results with reference to study objectives | **9-10** |
| Limitations | 19 | Discuss limitations of the study, taking into account sources of potential bias or imprecision. Discuss both direction and magnitude of any potential bias | **13** |
| Interpretation | 20 | Give a cautious overall interpretation of results considering objectives, limitations, multiplicity of analyses, results from similar studies, and other relevant evidence | **10** |
| Generalisability | 21 | Discuss the generalisability (external validity) of the study results | **10** |
| Other information | | | |
| Funding | 22 | Give the source of funding and the role of the funders for the present study and, if applicable, for the original study on which the present article is based | **Funding section** |

**Table S2. Maternal Early Obstetric Warning Score (MEOWS), showing the red (score 3), orange (score 2) and yellow (score 1) alerts for each individual category.**

| **Score** | **3** | **2** | **1** | **0** | **1** | **2** | **3** |
| --- | --- | --- | --- | --- | --- | --- | --- |
| **Temperature**  **(°C)** |  | < 35 | 35-35.9 | 36-37.4 | 37.5-37.9 | 38-38.9 | ≥ 39 |
| **Systolic BP**  **(mmHg)** | ≤ 69 | 70-79 | 80-89 | 90-139 | 140-149 | 150-159 | ≥ 160 |
| **Diastolic BP** |  |  | ≤ 49 | 50-89 | 90-99 | 100-109 | ≥ 110 |
| **Pulse** |  | < 40 | 40-49 | 50-99 | 100-109 | 110-129 | ≥ 130 |
| **Respiratory rate**  **(breaths per min)** | ≤ 10 |  |  | 11-19 | 20-24 | 25-29 | ≥ 30 |
| **AVPU** |  |  |  | Alert | Responds to voice | Responds to Pain | Unconscious |

**Table S3. Cox model report**

**Multivariate Cox regression**

|  | **Regression Coefficient** | **HR (95% CI for HR)** | **P value** |
| --- | --- | --- | --- |
|  |  |  |  |
| **Total MEOWS** | 0.097 | 1.10 (0.99 - 1.23) | 0.084 |
| **Sickle Cell group** | 1.123 | 3.4 (1.57 - 7.39) | 0.002* |

The p-value for all three overall tests (likelihood, Wald, and score) are significant, indicating that the model is significant. However, GLOBAL test is statistically significant indicating that hazard ratio is not constant over time.
